# Exogenous control over intracellular acidification: Enhancement via proton caged compounds coupled to gold nanoparticles and an alternative pathway with DMSO

**DOI:** 10.1016/j.dib.2015.12.032

**Published:** 2016-01-21

**Authors:** Marilena Carbone, Gianfranco Sabbatella, Simonetta Antonaroli, Hynd Remita, Viviana Orlando, Stefano Biagioni, Alessandro Nucara

**Affiliations:** a Dept. of Chemical Sciences and Technologies, University of Rome Tor Vergata, Via della Ricerca Scientifica, 1-00133 Rome, Italy; b Consorzio Interuniversitario Biostrutture e Biosistemi, Viale Medaglie d’Oro 305, 00136 Rome, Italy; c Dept. of Chemistry, University of Rome La Sapienza, P.le A. Moro, 00185 Rome, Italy; d Laboratoire de Chimie Physique, UMR 8000-CNRS, Bât. 349, Université Paris-Sud, 91405 Orsay, France; e Dept. of Biology and Biotechnology “Charles Darwin”, University of Rome La Sapienza, P.le A. Moro, 00185 Rome, Italy; f Dept. of Physics, University of Rome La Sapienza, P.le A. Moro, 00185 Rome, Italy; g Center for Life Nano Science, Istituto Italiano di Tecnologia, Viale Regina Elena 291, 00161 Rome, Italy

**Keywords:** Proton caged compounds, DMSO, Intracellular proton release

## Abstract

Proton caged compounds exhibit a characteristic behavior when directly dosed into cells or being coupled to gold nanoparticles prior to the dosing. When irradiated in the near ultraviolet region, they release protons that interact with intracellular HCO_3_^−^ to yield H_2_CO_3_. The dissociation of carbonic acid, then, releases CO_2_ that can be distinctively singled out in infrared spectra.

In the process of searching a pathway to augment the intracellular uptake of proton caged compounds, we probed the association of 1-(2-nitrophenyl)-ethylhexadecyl sulfonate (HDNS) with DMSO, an agent to enhance the membrane permeability. We found out a different UV-induced protonation mechanism that opens up to new conduits of employing of proton caged compounds. Here, we report the infrared data we collected in this set of experiments.

**Specifications Table**

**Table:** 

Subject area	***Biochemistry, Genetics and Molecular Biology***
More specific subject area	*Photochemistry, Photobiology*
Type of data	*Figures and Graphs*
How data was acquired	*Infrared spectroscopy, Bruker IFS66/VS interferometer, ultraviolet irradiation by deuterium discharge lamp (Acton Research Corporation) equipped with a band-pass FGUV11 filter (Thorlabs)*
Data format	*Raw and analyzed data*
Experimental factors	*NIH-3T3 cells are dosed with the proton caged compound HDNS and with DMSO to enhance the cells permeability*
Experimental features	*Infrared spectra are collected from NIH-3T3 cells upon irradiation with near UV-light and compared spectra of cells where the vectorization is achieved with gold nanoparticles*
Data source location	*Rome, Italy, 41.8536°N, 12.6033°E and 41.9033°N, 12.5158°E*
Data accessibility	*Data are with this article*

**Value of the data**

• These data exhibit the behavior of a proton caged compound into 3T3-NIH cells when dosed in association with the membrane cell permeability enhancer DMSO.
• The infrared spectra of the cells are taken after filtered ultraviolet light irradiation and show characteristic band variations.
• The band variation is different than in absence of DMSO, also compared to the vectorization with gold nanoparticles, opening up to new pathways of employing proton caged compounds.

## 1 Data

The first experiment to probe proton caged compounds (PCCs) as tools to manipulate and monitor the intracellular pH was performed by dosing the 1-(2-nitrophenyl)-ethylhexadecyl sulfonate (HDNS) to 3T3-NIH cells [1] and observing the effects on a single cell. PCCs yield one proton per molecule, therefore the intracellular proton release is related to the amount of PCCs that can be conveyed into the cells. An enhanced uptake can be obtained by vectorization of PCCs with gold nanoparticles (AuNPs) [2], or intervening on the cellular permeability, for instance, with DMSO. We have explored both pathways, the experiments with DMSO being antecedent the ones with AuNPs, because they do not require ad hoc synthesis of sulfur functionalized PCCs [3]. The outcome, though not straightforwardly applicable in the intracellular pH manipulation, is still quite interesting. Therefore, we report here the data we collected.

## 2 Experimental design, materials and methods

**Fig. 1:**
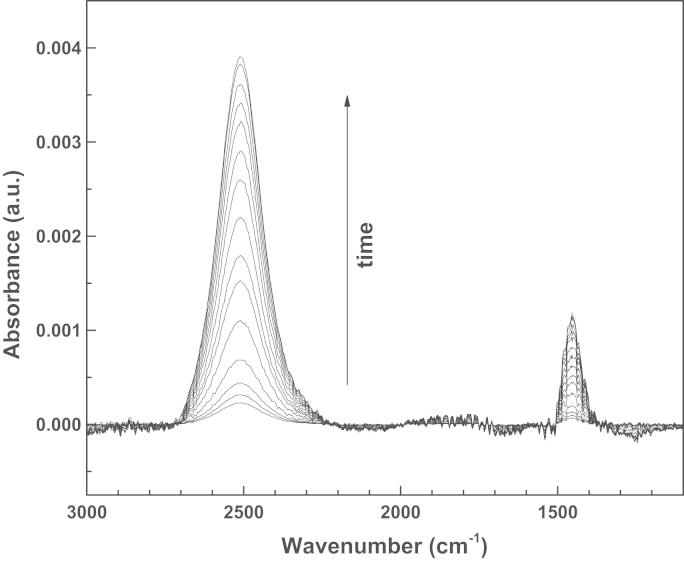
Infrared absorption spectra after irradiation of NIH-3T3 cells dosed with HDNS and DMSO: first data set. The arrow indicates the spectra evolution as a function of time.

**Fig. 2:**
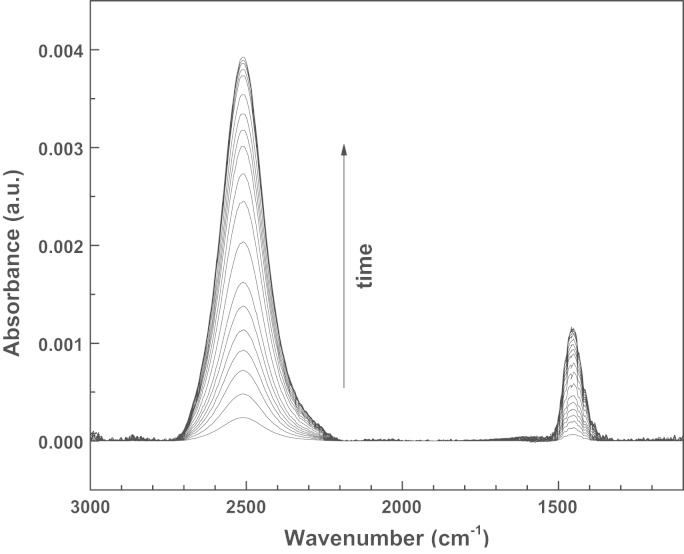
Infrared absorption spectra after irradiation of NIH-3T3 cells dosed with HDNS and DMSO: second data set. The arrow indicates the spectra evolution as a function of time.

The effects of the DMSO on the intracellular uptake of HDNS were monitored by dosing them simultaneously to 3T3-NIH cells and subsequently probing them by infrared spectroscopy upon irradiation with UV-light. More in detail, NIH 3T3 Swiss Albino Mouse Fibroblast cells (ECACC Catalog number 85022108) were cultured directly on UV-transparent CaF2 windows in Dulbecco׳s Modified Eagle Medium (DMEM) with HCO_3_^−^ (3.7 g/L) and supplemented with 10% fetal bovine serum up to full coverage. Afterwards, the cells were incubated with 3 mg HDNS and 2 μL DMSO in 3 mL in DMEM for 1 h. The amount of DMSO was chosen as the minimum amount to allow an increased membrane permeability [4], [5]. Afterwards the cell-coated window was transferred to the sample holder for liquids for collecting the infrared spectra, using a 12 μm Mylar spacer. The experimental setup was the same as the one used afterwards for the gold coupled PCCs [3] (a Bruker IFS66/VS interferometer, in transmission mode with a resolution of 2 cm^−1^). Infrared spectra were collected in the 3500–1000 cm^−1^ range for reference spectra and 3000–1000 cm^−1^ range for the cells. A few sequential infrared spectra of the cells were taken to verify their stability. Afterwards the samples were irradiated once for 1 min with near-UV light, by using a deuterium discharge lamp (Acton Research Corporation) equipped with a band-pass FGUV11 filter (Thorlabs) in the 275–375 nm. A few independent measurements were performed and they all provide similar outcome. Here we report two of them in Fig. 1, Fig. 2. The data are treated with the OPUS software for vector normalization and offset correction. Finally, they are normalized by the first spectrum after irradiation and exported as ASCII files. The spectra are taken at intervals of 2 min. Therefore, the whole datasets are taken in a time span of 30 (1st set) to 40 min (2nd set). In the second data set a saturation level may be appreciated.

**Fig. 3:**
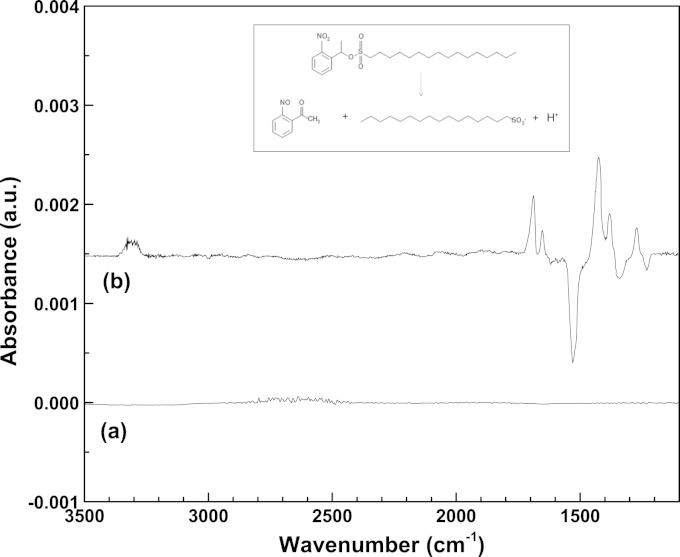
Infrared difference spectra before and after UV-irradiation: (a) DMSO, (b) HDNS in DMSO. The HDNS photodecomposition mechanism is shown in the inset.

The datasets are characterized by the arising of two bands which grow upon irradiation, one, rather large, centered at 2510 cm^−1^ and the other one narrower centered at 1452 cm^−1^. No contribution is found, instead, at 2343 cm^−1^ the typical value for the intracellular CO_2_. The data, however, are largely reproducible, and are not related to dead cells, since we had previously verified that in our experimental set up they do not provide any spectral variation as a function of irradiation and of time. Furthermore, we made checks to identify the new arising features, by comparing them to those of DMSO and HDNS in DMSO upon irradiation. In Fig. 3 the difference spectra are reported of DMSO (panel (a)) and HDNS M in DMSO (panel (b)) before and 5 min after UV irradiation. It can be observed that that DMSO is not affected by the irradiation. The PCC is obviously responsive to the irradiation and shows a number of positive and negative peaks that correspond to the breaking of the HDNS ester bond and the formation of a ketone and a sulfonic moiety, according to the mechanism illustrated in the inset of Fig. 3. In particular, the negative peaks at 1527 cm^−1^ and 1346 cm^−1^ are associated to the asymmetric and symmetric stretching of the NO_2_^−^ group, the negative peak at 1235 cm^−1^, to the sulfonic group. The positive peak at 1691 cm^−1^ can be associated to the newly formed ketone, whereas the peaks at 1424 cm^−1^, 1378 cm^−1^ and 1271 cm^−1^ are related to the cis and trans NO stretching [6]. The protons released in DMSO give rise to two new bands at 1652 cm^−1^ and 3320 cm^−1^ which can be associated to the bending and stretching of newly formed O–H^+^ interactions, as already observed for DMSO protonation by HCl [7]. The region around 2500 cm^−1^ is free both for DMSO and for HDNS in DMSO. This rules out that in the datasets of the cells, the increase of features intensity upon irradiation is the direct observation of processes solely related to the molecules themselves. The interpretation is therefore to be found in processes which may affect the cells as effect of the proton release. There are many possible intracellular contributions to the feature at 1452 cm^−1^, such as side chains and protonated side chains of several aminoacids and proteins as well as the Amide II band [8], [9].

The band at 2510 cm^−1^ is rarely observed. The most likely contributions can be associated to the dimer form of carboxylic acids, such as in solid state formic acid [10]. Furthermore, bands at ~2500 cm^−1^ are observed also for saturated carboxylic dimers [11]. This gives an indication of a direct protonation of aminoacids and proteins, rather than the reaction with HCO_3_^−^, when DMSO is used is association to a PCC. Alternatively, the band at 2510 cm^−1^ may be associated to processes which produce CSO [12] or SH [13].

However, in general, the arising of the features at 2510 cm^−1^ and 1452 cm^−1^ mainly points at a protonation mechanism induced by PCC and DMSO which affects the intracellular components in a more extended way as compared to the PCC/AuNPs or the PCC alone.
